# 7-Meth­oxy­indan-1-one

**DOI:** 10.1107/S1600536812040743

**Published:** 2012-10-03

**Authors:** Yuan Jay Chang, Kew-Yu Chen

**Affiliations:** a Department of Chemistry, Tung Hai University, 407 Taichung, Taiwan; b Department of Chemical Engineering, Feng Chia University, 40724 Taichung, Taiwan

## Abstract

In the title compound, C_10_H_10_O_2_, the 1-indanone unit is essentially planar (r.m.s. deviation = 0.028 Å). In the crystal, molecules are linked *via* C—H⋯O hydrogen bonds, forming layers lying parallel to the *ab* plane. This two-dimensional structure is stabilized by a weak C—H⋯π inter­action. A second weak C—H⋯π inter­action links the layers, forming a three-dimensional structure.

## Related literature

For the preparation of the title compound, see: Li *et al.* (2011 ▶). For applications of indanone derivatives, see: Borge *et al.* (2010 ▶); Cai *et al.* (2005 ▶); Cui *et al.* (2009 ▶); Fu & Wang (2008 ▶); Li *et al.* (2009 ▶); Sousa *et al.* (2011 ▶); Tang *et al.* (2011 ▶). For related structures, see: Ali *et al.* (2010*a* ▶,*b* ▶,*c* ▶,*d* ▶); Chen *et al.* (2011*a* ▶,*b* ▶). For C—H⋯O hydrogen bonds, see: Li *et al.* (2011*a* ▶,*b* ▶); Wang & Chen (2011 ▶); Xi *et al.* (2010 ▶).
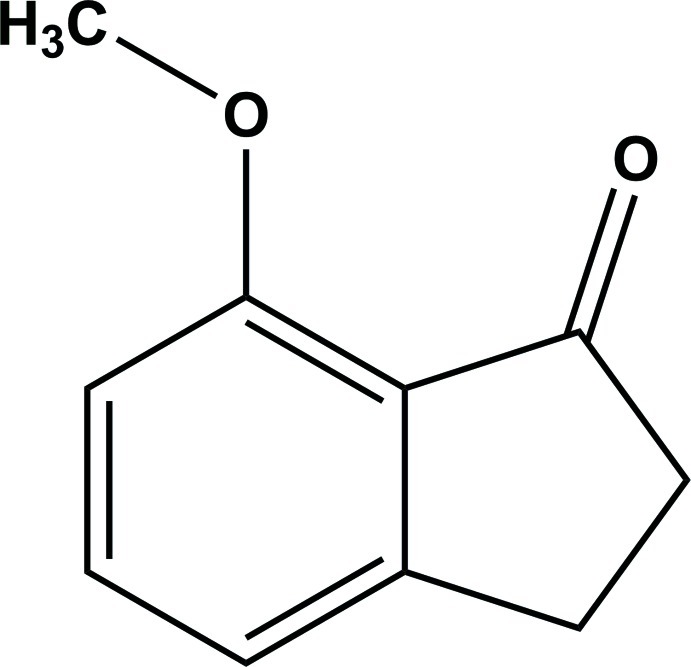


## Experimental

### 

#### Crystal data

C_10_H_10_O_2_
*M* *_r_* = 162.18
Orthorhombic, 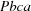

*a* = 8.5386 (7) Å
*b* = 10.4949 (9) Å
*c* = 18.8536 (16) Å
*V* = 1689.5 (2) Å^3^
*Z* = 8
Mo *K*α radiation
μ = 0.09 mm^−1^
*T* = 297 K
0.64 × 0.55 × 0.32 mm

#### Data collection

Bruker SMART CCD area-detector diffractometer
Absorption correction: multi-scan (*SADABS*; Bruker, 2001 ▶) *T*_min_ = 0.683, *T*_max_ = 1.000
8807 measured reflections
1663 independent reflections
1278 reflections with *I* > 2σ(*I*)
*R*_int_ = 0.032

#### Refinement

*R*[*F* ^2^ > 2σ(*F* ^2^)] = 0.037
*wR*(*F* ^2^) = 0.116
*S* = 1.02
1663 reflections
110 parameters
H-atom parameters constrained
Δρ_max_ = 0.20 e Å^−3^
Δρ_min_ = −0.13 e Å^−3^

### 

Data collection: *SMART* (Bruker, 2001 ▶); cell refinement: *SAINT* (Bruker, 2001 ▶); data reduction: *SAINT*; program(s) used to solve structure: *SHELXS97* (Sheldrick, 2008 ▶); program(s) used to refine structure: *SHELXL97* (Sheldrick, 2008 ▶); molecular graphics: *ORTEP-3 for Windows* (Farrugia, 1997 ▶); software used to prepare material for publication: *WinGX* publication routines (Farrugia, 1999 ▶).

## Supplementary Material

Crystal structure: contains datablock(s) I, global. DOI: 10.1107/S1600536812040743/zl2507sup1.cif

Structure factors: contains datablock(s) I. DOI: 10.1107/S1600536812040743/zl2507Isup2.hkl

Supplementary material file. DOI: 10.1107/S1600536812040743/zl2507Isup3.cml

Additional supplementary materials:  crystallographic information; 3D view; checkCIF report

## Figures and Tables

**Table 1:** Hydrogen-bond geometry (Å, °) *Cg*1 is the centroid of the C1/C5–C9 ring.

*D*—H⋯*A*	*D*—H	H⋯*A*	*D*⋯*A*	*D*—H⋯*A*
C3—H3*B*⋯O2^i^	0.97	2.60	3.5183 (18)	159
C7—H7*A*⋯O1^ii^	0.93	2.57	3.4802 (18)	167
C10—H10*B*⋯O1^iii^	0.96	2.59	3.486 (2)	156
C4—H4*A*⋯*Cg*1^iv^	0.97	2.80	3.6430 (16)	146
C10—H10*A*⋯*Cg*1^v^	0.96	2.82	3.6260 (16)	143

Symmetry codes: (i) 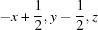; (ii) 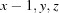; (iii) 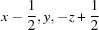; (iv) 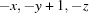; (v) 
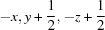
.

**Fig. 1.**
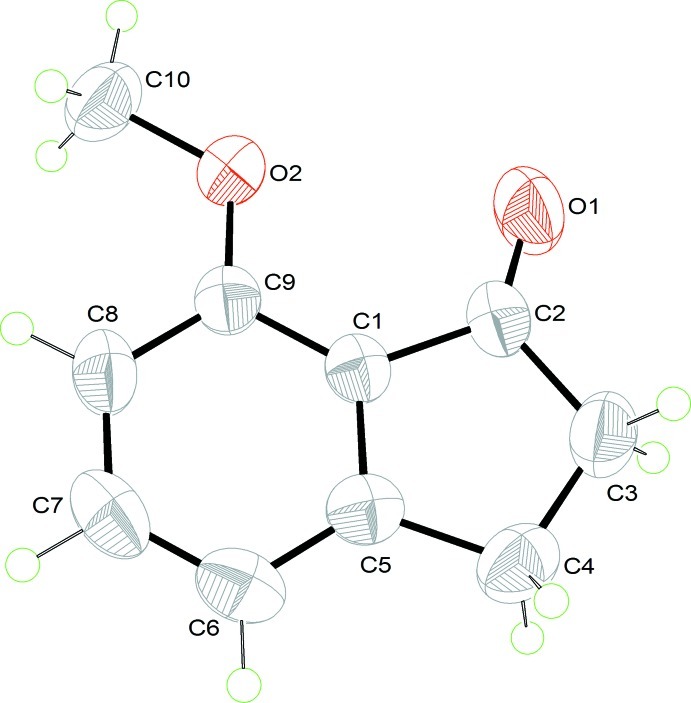
The molecular structure of the title compound, showing 50% probability displacement ellipsoids.

**Fig. 2.**
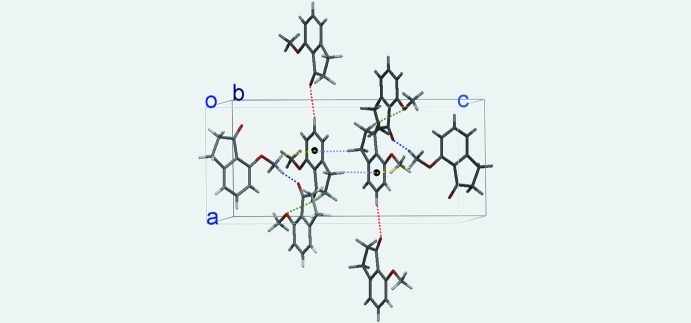
A section of the crystal packing of the title compound, viewed along the b axis. Blue, green and red dashed lines denote the intermolecular C10—H10B···O1, C3—H3B···O2 and C7—H7A···O1 hydrogen bonds, respectively. Yellow and purple dashed lines denote the intermolecular C10—H10A···π and C4—H4A···π hydrogen bonds, respectively. Cg1 (black circles) is the centroid of the C1/C5—C9 ring. For symmetry operators, see Table 1.

## supplementary crystallographic information

### Comment

Indanone and its derivatives are some of the most widely used organic compounds (Tang *et al.*, 2011). They are used as dyes and pigments (Cui *et al.*, 2009; Li *et al.*, 2009), intermediates in organic synthesis (Fu & Wang, 2008; Borge *et al.*, 2010) and exhibit a wide variety of biological activities (Sousa *et al.*, 2011). In addition, 1-indanones were important precursors in the regiospecific synthesis of 2-fluoro-1-naphthols (Cai *et al.*, 2005).

The molecular structure of the title compound is shown in Figure 1. The 1-indaneone moiety is essentially planar (r.m.s. deviation = 0.028 Å), which is consistent with previous studies (Chen *et al.*, 2011*a*,*b*; Ali *et al.*, 2010*a*,*b*,*c*,*d*). There are three different kinds of C—H···O (Li *et al.*, 2011*a*,*b*; Wang *et al.*, 2011; Xi *et al.*, 2010) hydrogen bonds (Table 1) in the crystal structure (Figure 2). In addition, C—H···π hydrogen bonds further stabilize the crystal structure (2.80 Å for the C4—H4A···*Cg*1 distance and 146° for the C4—H4A—*Cg*1^i^ angle; 2.82 Å for the C10—H10A···*Cg*1 distance and 143° for the C10—H10A—*Cg*1^ii^ angle; *Cg*1 is the centroid of the C1/C5—C9 ring; symmetry codes: (i): -*x*, 1 - *y*,- *z* (ii): -*x*, 1/2 + *y*, 1/2 - *z*).

### Experimental

The title compound was synthesized by the methylation of 7-hydroxyindan-1-one with methyl iodide (Li *et al.*, 2011). Colorless parallelepiped-shaped crystals suitable for the crystallographic study reported here were isolated over a period of six weeks by slow evaporation from a chloroform solution.

### Refinement

The C bound H atoms were positioned geometrically (C—H = 0.93–0.97 Å) and allowed to ride on their parent atoms, with *U*_iso_(H) = 1.2*U*_eq_(C).

### Crystal data

**Table:**

C_10_H_10_O_2_	*F*(000) = 688
*M**_r_* = 162.18	*D*_x_ = 1.275 Mg m^−^^3^
Orthorhombic, *P**b**c**a*	Mo *K*α radiation, λ = 0.71073 Å
Hall symbol: -P 2ac 2ab	Cell parameters from 3629 reflections
*a* = 8.5386 (7) Å	θ = 2.9–26.0°
*b* = 10.4949 (9) Å	µ = 0.09 mm^−^^1^
*c* = 18.8536 (16) Å	*T* = 297 K
*V* = 1689.5 (2) Å^3^	Parallelepiped, colorless
*Z* = 8	0.64 × 0.55 × 0.32 mm

### Data collection

**Table:**

Bruker SMART CCD area-detector diffractometer	1663 independent reflections
Radiation source: fine-focus sealed tube	1278 reflections with *I* > 2σ(*I*)
Graphite monochromator	*R*_int_ = 0.032
phi and ω scans	θ_max_ = 26.0°, θ_min_ = 2.2°
Absorption correction: multi-scan (*SADABS*; Bruker, 2001)	*h* = −10→10
*T*_min_ = 0.683, *T*_max_ = 1.000	*k* = −12→12
8807 measured reflections	*l* = −16→23

### Refinement

**Table:**

Refinement on *F*^2^	Secondary atom site location: difference Fourier map
Least-squares matrix: full	Hydrogen site location: inferred from neighbouring sites
*R*[*F*^2^ > 2σ(*F*^2^)] = 0.037	H-atom parameters constrained
*wR*(*F*^2^) = 0.116	*w* = 1/[σ^2^(*F*_o_^2^) + (0.0636*P*)^2^ + 0.2236*P*] where *P* = (*F*_o_^2^ + 2*F*_c_^2^)/3
*S* = 1.02	(Δ/σ)_max_ < 0.001
1663 reflections	Δρ_max_ = 0.20 e Å^−^^3^
110 parameters	Δρ_min_ = −0.13 e Å^−^^3^
0 restraints	Extinction correction: *SHELXL*, Fc^*^=kFc[1+0.001xFc^2^λ^3^/sin(2θ)]^-1/4^
Primary atom site location: structure-invariant direct methods	Extinction coefficient: 0.0068 (15)

### Special details

**Table:**

Geometry. All e.s.d.'s (except the e.s.d. in the dihedral angle between two l.s. planes) are estimated using the full covariance matrix. The cell e.s.d.'s are taken into account individually in the estimation of e.s.d.'s in distances, angles and torsion angles; correlations between e.s.d.'s in cell parameters are only used when they are defined by crystal symmetry. An approximate (isotropic) treatment of cell e.s.d.'s is used for estimating e.s.d.'s involving l.s. planes.
Refinement. Refinement of *F*^2^ against ALL reflections. The weighted *R*-factor *wR* and goodness of fit *S* are based on *F*^2^, conventional *R*-factors *R* are based on *F*, with *F* set to zero for negative *F*^2^. The threshold expression of *F*^2^ > σ(*F*^2^) is used only for calculating *R*-factors(gt) *etc*. and is not relevant to the choice of reflections for refinement. *R*-factors based on *F*^2^ are statistically about twice as large as those based on *F*, and *R*- factors based on ALL data will be even larger.

### Fractional atomic coordinates and isotropic or equivalent isotropic displacement parameters (Å^2^)

**Table:**

	*x*	*y*	*z*	*U*_iso_*/*U*_eq_	
O1	0.34168 (11)	0.54158 (11)	0.13950 (7)	0.0741 (4)	
O2	0.07222 (10)	0.67528 (9)	0.20144 (5)	0.0545 (3)	
C1	0.06609 (14)	0.50056 (11)	0.12296 (6)	0.0412 (3)	
C2	0.23519 (16)	0.47945 (13)	0.11460 (7)	0.0480 (3)	
C3	0.25583 (19)	0.36302 (14)	0.06788 (8)	0.0637 (4)	
H3A	0.3207	0.3835	0.0272	0.076*	
H3B	0.3057	0.2948	0.0943	0.076*	
C4	0.09387 (18)	0.32271 (14)	0.04396 (8)	0.0606 (4)	
H4A	0.0829	0.3318	−0.0070	0.073*	
H4B	0.0734	0.2348	0.0568	0.073*	
C5	−0.01569 (17)	0.41149 (12)	0.08242 (7)	0.0475 (3)	
C6	−0.17764 (18)	0.41110 (14)	0.08112 (8)	0.0610 (4)	
H6A	−0.2322	0.3522	0.0538	0.073*	
C7	−0.25596 (18)	0.49998 (16)	0.12127 (8)	0.0643 (5)	
H7A	−0.3649	0.5006	0.1207	0.077*	
C8	−0.17749 (16)	0.58882 (14)	0.16256 (7)	0.0560 (4)	
H8A	−0.2340	0.6470	0.1896	0.067*	
C9	−0.01507 (15)	0.59128 (12)	0.16371 (6)	0.0431 (3)	
C10	−0.0085 (2)	0.76805 (14)	0.24349 (8)	0.0662 (4)	
H10A	0.0664	0.8210	0.2674	0.099*	
H10B	−0.0729	0.7255	0.2779	0.099*	
H10C	−0.0730	0.8198	0.2134	0.099*	

### Atomic displacement parameters (Å^2^)

**Table:**

	*U*^11^	*U*^22^	*U*^33^	*U*^12^	*U*^13^	*U*^23^
O1	0.0400 (6)	0.0783 (8)	0.1039 (9)	0.0010 (5)	0.0001 (6)	−0.0157 (6)
O2	0.0529 (6)	0.0558 (6)	0.0548 (6)	0.0059 (4)	−0.0009 (4)	−0.0125 (4)
C1	0.0415 (7)	0.0426 (6)	0.0395 (6)	0.0022 (5)	0.0007 (5)	0.0071 (5)
C2	0.0418 (7)	0.0503 (7)	0.0520 (7)	0.0065 (6)	0.0016 (6)	0.0054 (6)
C3	0.0651 (9)	0.0637 (9)	0.0624 (8)	0.0227 (8)	0.0009 (7)	−0.0052 (7)
C4	0.0793 (11)	0.0469 (8)	0.0555 (8)	0.0084 (7)	−0.0041 (7)	−0.0038 (6)
C5	0.0565 (8)	0.0429 (7)	0.0432 (7)	−0.0009 (6)	−0.0041 (6)	0.0052 (5)
C6	0.0548 (9)	0.0610 (9)	0.0674 (9)	−0.0121 (7)	−0.0116 (7)	0.0017 (7)
C7	0.0380 (7)	0.0802 (11)	0.0747 (10)	−0.0061 (7)	−0.0015 (7)	0.0120 (8)
C8	0.0435 (8)	0.0664 (9)	0.0583 (8)	0.0090 (6)	0.0094 (6)	0.0041 (7)
C9	0.0440 (7)	0.0458 (7)	0.0393 (6)	0.0026 (5)	0.0020 (5)	0.0043 (5)
C10	0.0800 (10)	0.0591 (8)	0.0594 (9)	0.0161 (8)	0.0020 (8)	−0.0128 (7)

### Geometric parameters (Å, º)

**Table:**

O1—C2	1.2134 (17)	C4—H4B	0.9700
O2—C9	1.3560 (15)	C5—C6	1.383 (2)
O2—C10	1.4321 (16)	C6—C7	1.375 (2)
C1—C5	1.3948 (17)	C6—H6A	0.9300
C1—C9	1.4061 (17)	C7—C8	1.387 (2)
C1—C2	1.4692 (18)	C7—H7A	0.9300
C2—C3	1.517 (2)	C8—C9	1.387 (2)
C3—C4	1.515 (2)	C8—H8A	0.9300
C3—H3A	0.9700	C10—H10A	0.9600
C3—H3B	0.9700	C10—H10B	0.9600
C4—C5	1.5063 (19)	C10—H10C	0.9600
C4—H4A	0.9700		
			
C9—O2—C10	117.90 (11)	C6—C5—C4	127.63 (13)
C5—C1—C9	120.43 (12)	C1—C5—C4	111.55 (12)
C5—C1—C2	109.40 (11)	C7—C6—C5	118.33 (13)
C9—C1—C2	130.17 (12)	C7—C6—H6A	120.8
O1—C2—C1	127.87 (13)	C5—C6—H6A	120.8
O1—C2—C3	124.79 (13)	C6—C7—C8	122.01 (14)
C1—C2—C3	107.34 (12)	C6—C7—H7A	119.0
C4—C3—C2	106.97 (12)	C8—C7—H7A	119.0
C4—C3—H3A	110.3	C7—C8—C9	120.27 (13)
C2—C3—H3A	110.3	C7—C8—H8A	119.9
C4—C3—H3B	110.3	C9—C8—H8A	119.9
C2—C3—H3B	110.3	O2—C9—C8	124.74 (12)
H3A—C3—H3B	108.6	O2—C9—C1	117.13 (11)
C5—C4—C3	104.53 (11)	C8—C9—C1	118.14 (12)
C5—C4—H4A	110.8	O2—C10—H10A	109.5
C3—C4—H4A	110.8	O2—C10—H10B	109.5
C5—C4—H4B	110.8	H10A—C10—H10B	109.5
C3—C4—H4B	110.8	O2—C10—H10C	109.5
H4A—C4—H4B	108.9	H10A—C10—H10C	109.5
C6—C5—C1	120.81 (13)	H10B—C10—H10C	109.5
			
C5—C1—C2—O1	176.75 (14)	C1—C5—C6—C7	−0.6 (2)
C9—C1—C2—O1	−3.7 (2)	C4—C5—C6—C7	179.00 (13)
C5—C1—C2—C3	−3.15 (14)	C5—C6—C7—C8	−0.1 (2)
C9—C1—C2—C3	176.41 (12)	C6—C7—C8—C9	0.8 (2)
O1—C2—C3—C4	−175.29 (13)	C10—O2—C9—C8	0.29 (18)
C1—C2—C3—C4	4.62 (15)	C10—O2—C9—C1	−179.75 (11)
C2—C3—C4—C5	−4.24 (15)	C7—C8—C9—O2	179.03 (12)
C9—C1—C5—C6	0.42 (18)	C7—C8—C9—C1	−0.93 (19)
C2—C1—C5—C6	−179.97 (12)	C5—C1—C9—O2	−179.63 (10)
C9—C1—C5—C4	−179.21 (11)	C2—C1—C9—O2	0.84 (18)
C2—C1—C5—C4	0.41 (14)	C5—C1—C9—C8	0.33 (17)
C3—C4—C5—C6	−177.12 (14)	C2—C1—C9—C8	−179.19 (12)
C3—C4—C5—C1	2.47 (15)		

### Hydrogen-bond geometry (Å, º)

*Cg*1 is the centroid of the C1/C5–C9 ring.

**Table:**

*D*—H···*A*	*D*—H	H···*A*	*D*···*A*	*D*—H···*A*
C3—H3*B*···O2^i^	0.97	2.60	3.5183 (18)	159
C7—H7*A*···O1^ii^	0.93	2.57	3.4802 (18)	167
C10—H10*B*···O1^iii^	0.96	2.59	3.486 (2)	156
C4—H4*A*···*Cg*1^iv^	0.97	2.80	3.6430 (16)	146
C10—H10*A*···*Cg*1^v^	0.96	2.82	3.6260 (16)	143

Symmetry codes: (i) −*x*+1/2, *y*−1/2, *z*; (ii) *x*−1, *y*, *z*; (iii) *x*−1/2, *y*, −*z*+1/2; (iv) −*x*, −*y*+1, −*z*; (v) −*x*, *y*+1/2, −*z*+1/2.
